# Rethinking neurodegeneration: Placing disease in its physiological context

**DOI:** 10.1371/journal.pbio.3004087

**Published:** 2026-10-30

**Authors:** Joanna Clarke, Sarah Jäkel

**Affiliations:** 1 Public Library of Science, San Francisco, California, United States of America; 2 Public Library of Science, Cambridge, United Kingdom; 3 Department of Psychiatry and Psychotherapy, School of Medicine and Health, Technical University of Munich, Munich, Germany

## Abstract

Research into neurodegeneration is often confined to a single disease, cell type or mechanism. This Editorial highlights a new collection of articles that explore neurodegeneration within its physiological context, looking beyond diseased neurons to the surrounding tissue microenvironment.

## Teaser

Research into neurodegeneration is often confined to a single disease, cell type or mechanism. A new collection of articles explores neurodegeneration within its physiological context, looking beyond diseased neurons to the surrounding tissue microenvironment.

For decades, neurodegeneration has been framed around an apparently simple endpoint: neurons die. This focus has shaped how the field defines disease mechanisms, builds models and searches for therapies [1,2]. Yet, it also risks narrowing the question to what goes wrong within the neuron itself. In experimental models of amyotrophic lateral sclerosis (ALS; also known as motor neuron disease), genetic blockade of motor neuron death can preserve neuronal cell bodies while denervation and motor dysfunction still emerge [3], illustrating that neuronal death alone does not explain disease progression. In reality, neurons are embedded in one of the body’s most complex and dynamic tissue environments, which shapes how disease begins, spreads and progresses.

Moving beyond a purely neuron-centered view does not make neurons less important for neurodegenerative disease. The field has increasingly embraced a broader, multicellular perspective, with growing attention to neurodegeneration as a process shaped by glia, vasculature, immune signaling, metabolism, and aging [4]. Rather than displacing the neuron, this view asks us to consider neuronal vulnerability as something that emerges from the interplay between the neuron itself and the broader systems that sustain its function. Placing neurodegeneration in its physiological context therefore means asking not only why neurons die, but how changes in the surrounding tissue environment influence whether they remain resilient, become dysfunctional, or degenerate.

To explore this broader perspective, *PLOS Biology* presents a Collection of articles that look beyond diseased neurons, aiming to highlight the research underpinning our current knowledge of the physiological context of neurodegeneration and looking towards the future of neurodegenerative disease research. A key question for the field is why some neurons are affected by neurodegeneration and others are not. Each neurodegenerative disease affects a distinct area of the nervous system and type of neuron, suggesting that neuronal subtypes have specific vulnerabilities to disease; two Essays in this Collection investigate this idea of neuronal vulnerability, approaching the subject from very different angles. Wakatsuki and Araki ask why neurodegeneration affects cells in the central nervous system to a greater degree than those in the peripheral nervous system [5]. Using oxidative stress as a pathogenic mechanism, they examine how multilayered signaling thresholds could shape neuronal vulnerability. Dong Won Kim takes a different approach in his Essay, asking how neurons survive in neurodegenerative disease, rather than how they die [6]. The answer, he suggests, could be related to how well neurons are able to maintain their cellular subtype identity under stress.

Taking the idea of neuronal vulnerability in a more clinical direction, Gupta and colleagues look at the challenge of delivering therapeutic drugs to the right cells to treat neurodegenerative disease [7]. They highlight the idea of the ‘neurovascular unit’ and how its phenotype changes as disease progresses, and call for drug screening models that more accurately reflect the aged and diseased status of the brains that the therapies are being designed to treat. This sentiment is echoed by Liu and Ng in their call for organoid models of neurodegenerative disease to better represent the tissue microenvironment, calling for microglia and vascular cells to be included as standard [8].

The importance of non-neuronal cells within the nervous system and the role they might have in neurodegenerative disease is a recurring theme across this Collection. Linking a less well-studied type of neuronal support cell to possible disease mechanisms, Charles ffrench-Constant suggests a potential role for gray-matter oligodendrocytes in cognitive dysfunction in Alzheimer’s disease [9]. Echoing calls to remember the vasculature in disease models [7,8], Lewis and colleagues urge researchers to consider both the molecular and physiological phenotypes of blood vessels when investigating vascular dysfunction as a part of disease [10]. And, included in our online Collection content, Cheng and Ho ask the question of whether disease-associated microglia are protective or harmful in neurodegenerative disease [11], while Danzer and Sparrer examine data linking vaccinations to reduced dementia risk and ask if targeting viruses could offer a new avenue for treating neurodegenerative disease [12].

Taken together, the articles in this Collection demonstrate that the field has made significant progress by moving beyond a purely neuron-centered view of neurodegeneration, yet they also highlight the next challenge: avoiding a fragmented view in which each component of the tissue environment is studied in isolation. Fully understanding neurodegeneration in its physiological context will require moving from identifying affected cell types and systems to defining the relationships between them. We need to be asking how vascular dysfunction alters glial states, how glial responses influence synapses and myelin, how aging reshapes cellular resilience, and how stressed neurons feed back into their environment. Disease progression is therefore likely to depend not only on changes within individual cell types, but on how their relationships are rewired over time.

The same logic applies across neurodegenerative diseases themselves. Alzheimer’s disease, Parkinson’s disease, ALS, and other disorders are usually studied within disease-specific frameworks, but the physiological systems that fail during neurodegeneration do not necessarily respect these boundaries. These disorders may share underlying failures in the systems that maintain tissue homeostasis, even if those failures unfold differently across affected cells, circuits and brain regions. A more integrated view of neurodegeneration should therefore ask not only what distinguishes one disease from another, but what common failures of tissue physiology connect them.

Achieving this integration will require a new generation of models and methods. Many current approaches are still optimized to examine one cell type, pathway or disease feature at a time, whereas neurodegeneration unfolds in aged, multicellular and spatially organized tissue environments. Better models, combined with single-cell, spatial and longitudinal approaches, will be needed to link molecular and cellular changes to tissue architecture, circuit function and clinical progression.

The future of neurodegeneration research will depend on understanding disease not as the failure of isolated cells, but as the breakdown of relationships across cells, tissues and time. Placing neurodegeneration in its physiological context, as we aimed to do in this Collection of articles, offers the opportunity to reveal new points of intervention, potentially opening the way to therapeutic approaches that preserve resilience before degeneration becomes irreversible.
